# Progress in Otology

**Published:** 1901-09-28

**Authors:** 


### PROGRESS IN OTOLOGY.

(*Continued from page* 416.)

To Ballance,[13] who identifies himself largely with MacEwen in methods, is due the credit of immensely shortening the after period of a mastoid operation by division of the latter into two parts. These consist of 1. The operation for the removal of the disease; 2. The operation for the healing of the wound. What is new consists in the proposal to open up the wound in from ten days' to three weeks' time in order to transplant over the bone, now lined with a soft pile of velvety granulations, the large skin graft taken from the patient's arm or leg, and the special after-treatment of the second operation. The graft is papered over with gold leaf, which in due course is removed through the meatus. The entire operation from start to finish is exceedingly beautiful and artistic in the hands of Ballance, and many valuable hints may be gleaned from a perusal of his own description. Much of its success must depend upon attention to the minute details he gives, both of the work and the instruments, down to the dimensions of the razor employed for cutting the flap. His opening is large and roomy from the commencement, enabling a comprehensive view of the situation to be gained; the incision through the cartilaginous meatus is vertical, and is brought forwards and upwards to a level with the anterior root of the helix, the flap so made being stitched to the mastoid flap behind the ear. For burrowing in the bone a cavity bur is used, driven by a portable electro-motor, or, failing the bur, the square-edged gouges of Ballance's pattern. Other special and indispensable instruments are the graft-lifter, the stopper for pushing home both the graft and the gold leaf, and the suction pipette for withdrawing air and moisture from beneath them. Objections have been raised against the necessity of two anæsthesias as in Ballance's method, but this drawback may be said to be well compensated for by the great curtailment of the time taken up for the complete cure.

A review of aural progress is not now regarded as comprehensive without a glance at the surgery of the brain for disease that has originated in aural inflammation. When asked the other day whether abscess of the brain fell in the province of the aurist for surgical treatment, Mr. Ballance replied that he had seen very good operations performed upon the brain by aural surgeons and saw no reason why this work should be relegated to the pure surgeon. That gentleman's address upon this subject to the Otological Society's meeting in Edinburgh is a concise setting forth of the methods he practises and recommends after a long and valuable experience.[14] The anæsthetic should be chloroform, given warily in cases of cerebellar abscess owing to the liability of respiration to cease. The incision of the scalp is better in the form of a flap than a cross. It should be large and cut with its base downwards. The opening in the skull should also be free, the trephine being five-eighths of an inch in diameter, slightly conical, and with its teeth outside. When the disc of bone is removed, more bone should be cut away (for cerebral abscess) until the opening is enlarged to a parallelogram measuring one and three-quarter inches antero-posteriorly, and one inch vertically, but the lower edge of the parallelogram is marked by the trephine opening. Three-quarters of an inch of its antero-posterior extent should lie behind the centre of this aperture, and one inch in front. The lowest part of any temporo-sphenoidal abscess can be efficiently drained through this opening, and the tegmina, the usual source of infection, directly observed and removed. Of course if the abscess is above and behind or above and in front of this situation, more bone must be taken away in order to expose it. In cerebellar abscess the anterior edge of the trephine opening should touch the posterior border of the mastoid process. Its upper edge should be just below Reid's base line, thus the horizontal and vertical portions of the sigmoid sinus are avoided. This opening should be enlarged backwards and downwards until it is quite one and a quarter inches in antero-posterior and one inch in vertical extent, and may require to be still further extended, but this cannot be done with much advantage anteriorly. For the opening in the dura mater a flap is also preferable, the aperture being made with the knife, and the flap, with base sideways or even upwards, cut with blunt pointed scissors. The instrument recommended for incising either an immediately subcortical abscess or one the site of which is not obvious, and must be explored for, is a sharp-pointed, long and narrow knife, exception occurring only in certain cerebellar cases.

The trocar and canula may fail in several ways—by missing, passing clean through without tapping, or failing to penetrate the capsule of the abscess. If careful exploratory puncture fail to find the abscess, the finger inserted into the brain substance will almost infallibly detect the presence of a tense abnormal swelling. The difficulty comes in cases of flat oyster-like abscesses, lying beneath the cortex, and failing to be evacuated, when one or perhaps two abscesses already have been. After evacuation, the abscess may be entirely closed by the "waves of brain substance," if not, Ballance advocates irrigation with a weak antiseptic, *but not unless two drainage tubes have been so arranged as to insure the free escape of the fluid*; but he is in favour of irrigation only in very exceptional and chronic cases. As for the drainage tube the nature of which is of little consequence, when once introduced successfully it should not be disturbed for some time, and must be shortened or removed only as the cavity heals from the bottom. The wound is closed by replacing the flap, an aperture having previously been made in it corresponding to the opening in the brain, and suturing it in position with fine silkworm gut. The after treatment requires the closest personal attention; hernia cerebri—an evidence of sepsis, is best treated by sterilised antiseptic dressings, some protective being introduced between (perhaps gold leaf is the best) and slight elastic pressure being exerted in the later stages.

[13] Medico Chirurgical Society's Transactions, Vol. LXXXIV. [14] Reprint of Mr. Ballance's Paper, 1901.

